# Generating hematopoietic cells from human pluripotent stem cells: approaches, progress and challenges

**DOI:** 10.1186/s13619-023-00175-6

**Published:** 2023-09-01

**Authors:** Haiqiong Zheng, Yijin Chen, Qian Luo, Jie Zhang, Mengmeng Huang, Yulin Xu, Dawei Huo, Wei Shan, Ruxiu Tie, Meng Zhang, Pengxu Qian, He Huang

**Affiliations:** 1 Bone Marrow Transplantation Center, the First Affiliated Hospital, Zhejiang University School of Medicine, Zhejiang University, Hangzhou, 310012 China; 2 Liangzhu Laboratory, Zhejiang University Medical Center, 1369 West Wenyi Road, Hangzhou, 311121 China; 3 Institute of Hematology, Zhejiang University, Hangzhou, 310012 China; 4 Zhejiang Province Engineering Laboratory for Stem Cell and Immunity Therapy, Hangzhou, 310012 China; 5 Center for Stem Cell and Regenerative Medicine and Bone Marrow Transplantation Center of the First Affiliated Hospital, Zhejiang University School of Medicine, Hangzhou, 310058 China

**Keywords:** Human pluripotent stem cells, Hematopoietic differentiation, Hematopoietic stem cells, Blood cells

## Abstract

Human pluripotent stem cells (hPSCs) have been suggested as a potential source for the production of blood cells for clinical application. In two decades, almost all types of blood cells can be successfully generated from hPSCs through various differentiated strategies. Meanwhile, with a deeper understanding of hematopoiesis, higher efficiency of generating progenitors and precursors of blood cells from hPSCs is achieved. However, how to generate large-scale mature functional cells from hPSCs for clinical use is still difficult. In this review, we summarized recent approaches that generated both hematopoietic stem cells and mature lineage cells from hPSCs, and remarked their efficiency and mechanisms in producing mature functional cells. We also discussed the major challenges in hPSC-derived products of blood cells and provided some potential solutions. Our review summarized efficient, simple, and defined methodologies for developing good manufacturing practice standards for hPSC-derived blood cells, which will facilitate the translation of these products into the clinic.

## Background

**Fig. 1:**
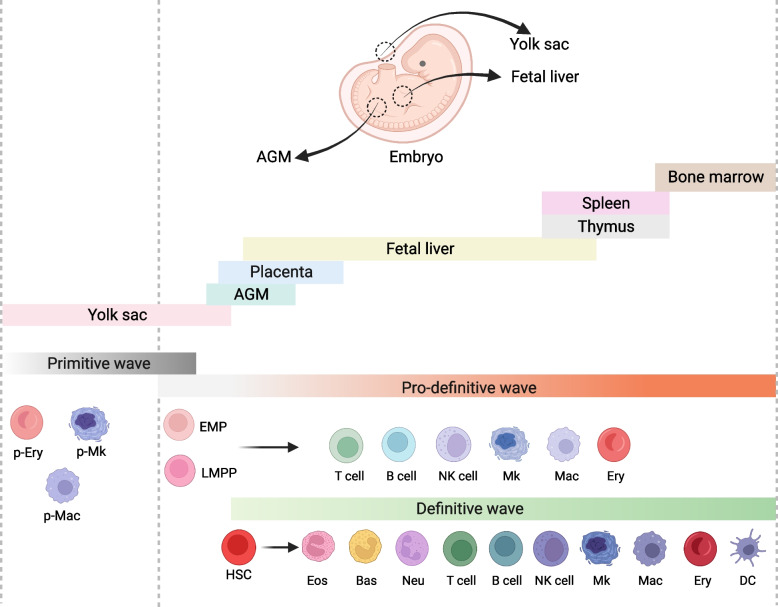
Schematic representations of embryonic hematopoiesis in vivo. There are 3 waves of hematopoietic cell generation, including primitive, pro-definitive, and definitive HSC. Among them, the primitive hematopoietic cells, including erythrocytes, megakaryocytes and macrophages, emerge in the extraembryonic yolk sac blood islands; the pro-definitive wave of hematopoiesis occurs in the yolk sac and generates definitive erythro-myeloid progenitors (EMPs) and lymphoid-primed progenitors (LMPPs) in the yolk sac; the definitive HSCs arise in the intraembryonic aorta–gonad–mesonephros region (AGM) and migrate into the fetal liver for maturation, expansion and differentiation, then initiate mobilization to the bone marrow to supply life-long hematopoiesis

Blood cells are the most abundant cell in mammals that play an indispensable role in survival and development. Hematopoiesis originates from the mesoderm and occurs across distinct anatomical sites and time (Fig. 1) (Huber et al. 2004). The primitive hematopoiesis first occurs in extraembryonic yolk sac (YS) “blood island” (murine embryonic (E) day 7.5, human 3–4 weeks) to generate transitory megakaryocytes (MKs), macrophages, and nucleated erythroblasts (Orkin and Zon 2008). These primitive erythroid cells express embryonic globin and undergo enucleation in the circulation (Kingsley et al. 2004). At murine E8.5-9.5, multipotent erythro-myeloid progenitors (EMPs) and lymphoid progenitors are generated, and tissue-resident immune cells (like B-1 cells (Baumgarth 2017; Hayakawa et al. 1985), Vγ3-T cells (Gentek et al. 2018; Park et al. 2020; Yoshimoto et al. 2012), macrophages (Gomez Perdiguero et al. 2015), and definitive erythrocytes) arise independently of hematopoietic stem cells (HSCs) in multiple waves of hematopoiesis (Dzierzak and Bigas 2018; Kobayashi and Yoshimoto 2023). In the definitive hematopoietic wave, also called HSC-dependent hematopoiesis, the long-term HSCs (LT-HSCs) are generated from the hemogenic endothelium (HE) of aorta-gonad mesonephros (AGM) region through endothelial-to-hematopoietic transition (EHT) (murine E10.5, human 5–6 weeks), as well as in the placental labyrinth, vitelline and umbilical arteries (Canu and Ruhrberg 2021; Lancrin et al. 2009; Medvinsky and Dzierzak 1996; Robin et al. 2009). Furthermore, mechanical forces from aortic blood flow further trigger definitive LT-HSC specification (Adamo et al. 2009; Lundin et al. 2020; North et al. 2009; Wang et al. 2011). These LT-HSCs migrate to fetal liver (FL), spleen, thymus, and finally bone marrow (BM). LT-HSCs reside in BM and support all mature blood cells (lymphoid cells, myeloid cells, erythrocytes and MKs) throughout adult life (Medvinsky et al. 2011).

Hematopoietic stem cell transplantation remains the most effective cellular replacement therapy for many life-threatening congenital and acquired hematopoietic disorders, including sickle cell disease, myelodysplastic syndrome, deadly leukemia, and so on (de Witte et al. 2017; Lv and Huang 2012; Vermylen 2003). Moreover, adaptive transfusion of terminal differentiated blood cells is also an effective therapy for various diseases. Such as red blood cells (RBCs) for benign or malignant anemia, chimeric antigen receptor (CAR)-engineered killer cells for cancers and autoimmune disorders (Labanieh et al. 2018; Orvain et al. 2021; Shah et al. 2015; Xie et al. 2020). But the clinical applications of these therapies are also limited by the availability of primary mature blood cells. Hence, it is important to find alternative sources and expand sufficient HSCs as well as terminally differentiated blood cells in vitro for heavy demand of clinical medicine.

Human pluripotent stem cells (hPSCs), including embryonic stem cells (ESCs) and induced pluripotent stem cells (iPSCs), are potential novel sources for off-the-shelf clinically applicable hematopoietic cell generation. ESCs were first isolated from inner cell mass of mice in 1981 (Evans and Kaufman 1981). Human ESCs were obtained from the blastocyst in 1998 (Thomson et al. 1998). In 2006, Takahashi and Yamanaka successfully generated iPSCs from mouse fibroblasts through ectopic expression of Sox2, Oct3/4, c-Myc, and Klf4 (Takahashi and Yamanaka 2006). This advance provided a theoretically unlimited source of patient-specific stem cells. Last year, Deng’s group successfully generated human iPSCs in a chemical-induced method (Guan et al. 2022), which boosted the iPSC-derived cell therapy into a new stage. hPSC-derived hematopoietic cells have tremendous potential for immunotherapies, transfusion and transplantation. To date, almost all kinds of hematopoietic cells can be generated from hPSCs, and some of them reached clinical grade (like natural killer (NK) cells and platelets) (Table 1).

**Table 1:** Current strategies for the differentiation of functional hematopoietic cells from hPSCs.

Cell source	Methods	Main cytokines	Products	Functional Assessment		References
Human ESCs	EB formation	SCF, FLT-3L, IL-3, IL-6, G-CSF, BMP-4	HSPCs	Self-renewal; multiple lineage progenitors in vitro	Chadwick et al. 2003	
Human ESCs	Co-culture with OP9, S17 or MS5	SCF, FLT-3L, IL-7, IL-3	HSPCs	Self-renewal; multiple lineages (including lymphoid lineage) in vitro		Vodyanik et al. 2005
Human ESCs and iPSCs	Serum and feeder-free; 3D microcarrier and EB formation	VEGF, BMP-4, bFGF	HSPCs	Ten-times higher yields of hematopoietic cells than 2D culture; multiple lineage progenitors in vitro		Lu et al. 2013
Human iPSCs	EB formation; 3D hemanoids	IL-3, M-CSF	HSPCs	Myeloid progenitors in vitro		Ackermann et al. 2021
Human ESCs and iPSCs	Co-culture with OP9; HE formation	SCF, IL-3, IL-6, TPO	HSPCs	Hemogenic endothelial progenitor identification; multiple lineage progenitors in vitro		Choi et al. 2012
Human ESCs and iPSCs	EB formation; HE formation	Seven TFs: ERG, HOXA5, HOXA9, HOXA10, LCOR, RUNX1 and SPI1	HSPCs	Multiple lineages (including lymphoid lineage) in vivo		Sugimura et al. 2017
Human iPSCs	Co-injection with OP9; teratoma formation	In vivo	HSPCs	Reconstitute immune system (including lymphoid lineage) in primary and secondary recipients		Amabile et al. 2013
Human iPSCs	HSPCs co-culture with OP9-DLL1	FLT-3L, IL-7, IL-21	T cells	Early memory phenotype and exert anti-viral/anti-tumor activity in vivo		Kawai et al. 2021
Human iPSCs	HSPCs co-culture with ATOs and MS5	SCF, FLT-3L, IL-7, TPO	T cells	Naïve phenotype and show anti-tumor in vitro and in vivo		Montel-Hagen et al. 2019; Seet et al. 2017
Human iPSCs	HSPCs co-culture with OP9-DLL1	SCF, FLT-3L, IL-7, IL-15	CAR-T cells	Innate γδ-T phenotype and exert anti-tumor activity in vivo		Themeli et al. 2013
Human iPSCs	Serum and feeder-free	SCF, FLT-3L, IL-7, TPO, SDF-1α, SB203580	CAR-T cells	TCR-dependent function and exert anti-tumor activity in vitro and in vivo		Iriguchi et al. 2021
Human ESCs	HSPCs co-culture with AFT024	SCF, FLT-3L, IL-7, IL-15, IL-3	NK cells	Express inhibitory and activating receptors and lyse tumor cells in vitro and in vivo		Woll et al. 2005; Woll et al. 2009
Human ESCs and iPSCs	HSPCs co-culture with EL08-1D2 or feeder-free; NK cells co-culture with aAPCs	SCF, FLT-3L, IL-7, IL-15, IL-3	NK cells	Exert anti-tumor activity in vitro and in vivo		Hermanson et al. 2016; Knorr et al. 2013
Human iPSCs	NK cells co-culture with aAPCs	SCF, FLT-3L, IL-7, IL-15, IL-3	CAR-NK cells	Mesothelin-specific CAR and anti-ovarian cancer with less toxicity in vivo		Li et al. 2018
Human iPSCs	HSPCs co-culture with MS5	SCF, FLT-3L, IL-7, IL-15, IL-3	B cells	VDJ rearrangement in H chain and express surface IgM in vitro		Carpenter et al. 2011; French et al. 2015
Human ESCs	Serum and feeder-free	SCF, IL-11, TPO	MKs and platelets	About 40% platelets in vitro and contribute to developing thrombi in vivo		Lu et al. 2011
Human ESCs and iPSCs (GATA1, FLI1 and TAL1)	Serum and feeder-free	SCF, IL-1β, TPO	MKs and platelets	About 5 platelets per MK in vitro		Moreau et al. 2016
Human ESCs and iPSCs (c-MYC, BMI1, BCL-XL)	Serum and feeder-free	SCF, TA-316, KP-457, SR1, GNF-351	MKs and platelets	About 70–80 platelets per MK in vitro and show turbulence in thrombopoiesis in vivo		Ito et al. 2018
Human ESCs and iPSCs	HSPCs co-culture with MS5	SCF, IL-3, IL-6, TPO, EPO	Erythroblasts	Express α, ζ, ɛ, γ hemoglobin and enucleation		Dias et al. 2011; Klimchenko et al. 2009
Human ESCs and iPSCs	Serum and feeder-free	SCF, IL-3, EPO	Erythroblasts	Express α, β, ɛ, ζ, γ hemoglobin and enucleation		Lapillonne et al. 2010; Lu et al. 2008
Human ESCs and iPSCs	Serum and feeder-free	IL-3, M-CSF	Monocytes/Macrophages	Possess highly phagocytic function and release cytokines for stimulation in vitro; Polarize to M1 or M2 phenotype and improve liver fibrosis in vivo		Pouyanfard et al. 2021; van Wilgenburg et al. 2013
Human iPSCs	Feeder-free	SCF, IL-3, M-CSF, GM-CSF, IGF-1	CAR-macrophages	Secrete cytokines, polarize to M1 phenotype, and anti-tumor in vivo		Zhang et al. 2020
Human ESCs	HSPCs co-culture with OP9	SCF, FLT-3L, IL-3, IL6, TPO, G-CSF	Neutrophils	Produce superoxide, phagocytose, engage in bactericidal activity, and chemotaxis in vitro; Recruit immune cells and against bacterial infection in vivo		Miyauchi et al. 2021; Yokoyama et al. 2009
Human ESCs and iPSCs	Serum and feeder-free	SCF, IL-3, IL5	Eosinophils	Express peroxidase and exert tumor killing capacity in vivo		Lai et al. 2021
Human iPSCs	Serum and feeder-free	SCF, IL-3, IL-6, LDL	Mast cells	Express specific markers and degranulate in response to IgE/anti-IgE and substance P in vitro		Luo et al. 2022

*Abbreviations*: *hPSCs* Human pluripotent stem cells, *ESCs* Embryonic stem cells, *EB* Embryoid body, *SCF* Stem cell factor, *FLT-3L* FMS-related tyrosine kinase 3 ligand, *IL-3* Interleukin-3, *G-CSF* Granulocyte colony stimulating factor, *BMP-4* Bone morphogenetic protein-4, *HSPCs* Hematopoietic stem/progenitor cells, *iPSCs* Induced pluripotent stem cells, *3D* Three-dimensional, *VEGF* Vascular endothlial growth factor, *bFGF* Basic fibroblast growth factor, *2D* Two-dimensional, *M-CSF* Macrophage colony stimulating factor, *HE* Hemogenic endothelium, *TPO* Thrombopoietin, *ATOs* Artificial thymic organoids, *CAR-T* Chimeric antigen receptor T, *SDF-1α* Stromal cell-derived factor-1 alpha, *aAPCs* Artifical antigen-presentingg cells, *MKs* Megakaryocytes, *SR1* StemRegenin 1, *EPO* Erythropoietin, *GM-CSF* Granulocyte-macrophage colony stimulating factor, *IGF-1* Insulin-like growth factor-1, *LDL* Low density lipoprotein

In this review, we will summarize the latest progresses of hematopoietic cell differentiation from hPSCs, discuss the remaining challenges and try to provide some potential solutions.

## Directed differentiation of HSCs from hPSCs

**Fig. 2:**
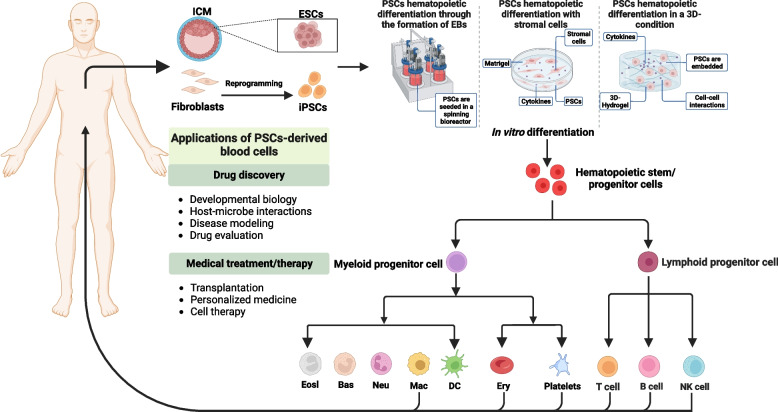
Overview of current methods of inducing PSCs into blood cells in vitro and potential applications of PSCs-derived blood cells. Several differentiation methods have been developed to derive blood cells from PSCs in vitro at present: through the formation of EBs, co-culture with stromal cells and induced hematopoietic differentiation in a 3D-condition. The generated blood cells could be utilized in certain fields: (1) Drug discovery: developmental biology, host-microbe interactions, disease modeling and drug evaluation; (2) Medical treatment/therapy for clinical use, such as transplantation, personalized medicine and cell therapy

As the top class of hematopoietic hierarchy, HSCs can generate all kinds of functional mature blood cells and update the old ones in circulation (Orkin and Zon 2008). Since the limited sources of primary human HSCs, and poor proliferation of HSCs in culture, an alternative HSC source is required for clinical transplantation and hematopoietic researches (Boitano et al. 2010; Fares et al. 2014; Walasek et al. 2012). hPSCs is an optimal tool to produce sufficient HSCs due to their self-renew capacity and differentiation potential (Takahashi and Yamanaka 2006; Thomson et al. 1998). To date, several differentiation methods have been well-developed to derive hematopoietic cells from hPSCs (Fig. 2).

### Generation of hematopoietic stem/progenitor cells (HSPCs) from hPSCs in vitro

Hematopoietic cells were first generated from ESCs in vitro by using embryoid bodies (EBs) system in semisolid media containing 10% fetal calf serum (Wiles and Keller 1991). EBs are multicellular three-dimensional (3D) structures spontaneously formed by hPSCs and consisted of 3 germ layers that mimic early embryo development to provide a microenvironmental signal during induced differentiation process (Chadwick et al. 2003; Lin and Chang 2008; Lu et al. 2013). However, this method has limited efficiency in producing hematopoietic cells which depends on the size and quality of the formed EBs (Ding et al. 2023). Of note, EB-derived hematopoietic cells showed primitive features with erythroblasts carrying γ-globin, indicating that it is difficult to generate definitive hematopoietic cells by EBs only.

The other method widely used is coculture of hPSCs with stromal cell lines, such as OP9 (Vodyanik et al. 2005), S17 (Kaufman et al. 2001), MS5 (Vodyanik et al. 2005) and so on, which could produce cytokines, such as Activin-A and bone morphogenetic protein (BMP) favorable for hematopoietic differentiation of hPSCs. To promote definitive hematopoietic development, Woods et al. developed an optimized differentiation protocol for the generation of precursors of hematopoietic lineages and primitive hematopoietic cells from hPSCs by combining these two ways. They plated the dissociated EBs on OP9 feeder cells and efficiently generated multipotent hematopoietic progenitor cells up to 84% CD45^+^ cells but unfortunately with limited engrafting ability in transplanted mice (Woods et al. 2011).

In addition to the methods above, monolayer-based serum-free differentiation approaches are also available to produce large-scale hematopoietic cells from hPSCs. hPSCs are plated on extracellular matrices, such as Matrigel, Vitronectin, Tenascin C, and collagen, and different combinations of cytokines and small molecules are added at suitable stages (Demirci et al. 2020; Ditadi and Sturgeon 2016; Niwa et al. 2011; Shen et al. 2021; Uenishi et al. 2014). For example, CHIR99021, ActivinA, Y-27,632, basic fibroblast growth factor (bFGF) and BMP-4 were used to induce the differentiation from hPSCs to mesodermal cells, and then vascular endothelial growth factor (VEGF), stem cell factor (SCF), thrombopoietin (TPO), FMS-related tyrosine kinase 3 ligand (FLT-3L), interleukin-3 (IL-3), IL-6, and StemRegenin 1 (SR1) were added to induce subsequent hematopoietic differentiation. Incremental improvements in hPSC-derived HSPCs may be possible by combining several of the above-mentioned approaches.

In the above methods, hPSCs are induced in a two-dimensional (2D) environment which is much more simplistic compared with the intricate environment in vivo. This fails to create a micro-environment capable of mimicking the in vivo hPSC differentiation. Therefore, several groups, including ours, have thus far established 3D systems to improve the efficiency of hematopoietic differentiation from PSCs (Ackermann et al. 2021; Ma et al. 2021; Shan et al. 2021; Wu et al. 2023). We successfully established 3D self-assembling peptide hydrogel combined with hematopoietic cytokines to promote the differentiation of mouse PSCs into clusters of hematopoietic cells. And the hematopoietic cells obtained from the 3D system had been proven to have the ability of hematopoietic reconstitution in short-term (Shan et al. 2021). A similar 3D culture method was also reported by Lu et al., they established a 3D microcarrier system and showed that the microcarrier platform could generate ten times higher yields of hematopoietic cells from hPSCs compared with conventional 2D culture (Lu et al. 2013). These results manifested the superiority of 3D culture system as an advanced technology for hPSC-derived hematopoietic differentiation. Thus it would be a revolutionary approach to solve the problems we faced now, including laborious, time-consuming, and too expensive for the generation of HSPCs from hPSCs.

### Derivation of HE from hPSCs

Vascular endothelial cells line the walls of all blood vessels. During embryonic development, a specialized subset of vascular endothelial cells, termed HE, arise at YS, placenta and AGM, giving rise to definitive hematopoiesis. HE at YS and placenta can generate short-term progenitors, like EMPs and lymphoid progenitors, while HE at AGM gives rise to LT-HSCs (Cumano et al. 2001; Wu and Hirschi 2021). At AGM, HE undergoes EHT to obtain specific lineage characteristics: upregulation of hematopoietic cell markers CD43 and CD45, while downregulation of endothelial makers, like CD144 and CD31, namely conduct a transition of endothelial cells to a hematopoietic cell fate to become HSCs (Bertrand et al. 2010; Guibentif et al. 2017; Velten et al. 2017; Zhou et al. 2016). Inspired by the EHT process, several groups have established approaches to differentiate HE from hPSCs to generate functional HSPCs and blood cells in vitro (Choi et al. 2012; Ditadi et al. 2015; Garcia-Alegria et al. 2018; Kennedy et al. 2012; Vargas-Valderrama et al. 2022). Choi et al. showed that definitive hematopoietic cells can be obtained from hPSC-derived HE progenitors and equipped with an enhanced myeloid and erythroid lineage potential. More importantly, they can be identified by the expression of precise markers: CD144^+^ CD73^−^ CD235a^−^/CD43^−^, which seems to be a meaningful panel to distinguish HE from hPSCs for further study (Choi et al. 2012). In addition to this study, HE have also been identified by other groups: co-expression of CD144, CD31, CD34, CD44, CD117, CD201, and lack of CD43, CD45, CD73 (Kissa and Herbomel 2010; Lange et al. 2021).

As key directors during hematopoietic development, transcription factors (TFs) have been used in ex vivo differentiation approaches based on their discovered functions during vertebrate ontogeny. Therefore, some of them have been used to enhance HE differentiation in several approaches. Lange et al. established an efficient, defined, and stable method based on TF-mediated hemato-endothelial inducible forward programming of human iPSCs and produced a large number of hematopoietic endothelial progenitor cells by enforced expression of SCL, LMO2, GATA2, and ETV2, which can generate hematopoietic progenitor cells through EHT (Lange et al. 2020). In contrast to other approaches, their protocol was fully inducible and required overexpression of TFs only for a short period to rapidly generate numerous highly pure HE progenitors with no influence on the production of hematopoietic progenitor cells. Ectopic expression of these TFs enhanced the stem cell properties of hematopoietic progenitor cells. However, long-term engraftment and multi-lineage reconstitution were not achieved. By in vivo screening of TFs for hematopoietic progenitor specification from HE, Daley’s group identified seven TFs and obtained hPSC-derived engraftable HSPCs in vivo by expressing these TFs, including ERG, HOXA5, HOXA9, HOXA10, LCOR, RUNX1 and SPI1, that together can confer HSC-like engraftment, self-renewal, and multi-lineage capacity. Each of the identified TFs in this study has been reported playing a role in HSC development, maintenance of long-term HSCs, or lineage commitment. Despite exciting results, the molecular mechanism between these engineered cells and bona fide HSCs still needs to be explored (Sugimura et al. 2017).

Collectively, these methods suggested that generation of hPSC-derived functional HSC through EHT is becoming more feasible, whereas our ultimate goal should be to obtain bona fide transgene-free HSCs for therapeutic strategies in genetic blood disorders and research.

### HSC generation from teratomas in vivo

In vitro generation of HSPCs has been widely reported, however, it is difficult to generate truly functional HSCs from hPSCs. What’s more, it could even exhibit abnormal hematopoiesis, let alone for transplantation. One of the reasons is that it is hard to reproduce the microenvironment necessary for hematopoiesis like embryonic development. Additionally, the whole cell types in HSC niche are utterly unknown, which makes microenvironment simulation more difficult. For these reasons, Amabile et al. utilized a new strategy by injecting human iPSCs into NSD mice to generate CD45^+^ hematopoietic progenitors via teratoma formation, which is a kind of benign tumor containing differentiated tissues of all three germ layers. They found that these CD34^+^CD45^+^ cells isolated from teratoma parenchyma possessed multi-lineage reconstitution capability as well as the differentiated myeloid and lymphoid progeny (Amabile et al. 2013). This is the first study showing a novel in vivo system that hPSCs in a physiologic environment have the ability of multi-lineage reconstitution.

In the same year, Suzuki et al. reported a similar method that co-injection of iPSCs with OP9 cells into NOD/SCID mice in the presence of exogenous hematopoietic cytokines. They showed that the generated HSCs could transfer into BM from the formed teratomas and enable long-term reconstruction and multilineage of the hematopoietic system. Particularly, leukemia and other tumors were not found in recipients after transplantation (Suzuki et al. 2013). Although similar works were accomplished by two independent groups, Suzuki et al. investigated the optimal conditions for in vivo induction of HSCs using Lnk^−/−^ mice. Moreover, they demonstrated that gene correction in X-SCID mice-derived iPSCs could overcome immunodeficiency by generation of T lymphocytes through teratoma formation. Based on this in vivo system, their team modified it for further optimization and improvement in the year 2017. By overexpression of Gfi1b, c-Fos, and Gata2, they showed the optimized induction system can generate functional LT-HSCs with high efficiency. More importantly, the authors further demonstrated that teratomas could be transplanted and cryopreserved (Tsukada et al. 2017).

Collectively, teratoma hematopoiesis in vivo is a promising model for production of hPSCs-derived HSCs, providing new technologies that could be useful for transplantation in future clinical applications.

## Directed differentiation of lymphoid cells from hPSCs

### T cells

During T lymphopoiesis in thymus, thymus-seeding progenitors firstly rearrange T cell receptor (TCR) β locus (Diversity and Joining regions, DJ regions) to generate early thymic progenitors and pre-T cells, then following the rearrangement of TCR β locus (Variable region, V region) or TCR γδ locus to generate αβ-commitment cells and γδ-commitment cells, respectively. During the differentiation of αβ-T cells, CD4^+^ immature single-positive cells differentiate into CD4^+^ and CD8^+^ double-positive cells under the rearrangement of TCR α locus (VJ regions), and finally become mature CD4^+^ or CD8^+^ single-positive T cells (Themeli et al. 2015).

In 2009, Timmermans et al. *de novo* regenerated functional CD3^+^ TCR^+^ T cells in vitro that showed a physiological T cell developmental process in culture system (Timmermans et al. 2009). Simply, they first cocultured human ESCs and OP9 cell line to generate hematopoietic precursor cells through hematopoietic zones, which were morphologically similar to embryonic blood island. Then they replated these hematopoietic zones into OP9-DLL1 cell line to promote T cell differentiation. They found that these artificial T cells were derived from CD34^high^ CD43^low^ population of hematopoietic zones and were polyclonal. Moreover, these T cells presented CD27^+^ CD1a^−^ mature phenotype and responded to PHA. After then, several studies have successfully generated T cells derived from iPSCs by similar methods. However, these iPSC-derived T cells are not truly primary human T cells, because they may lack surface markers like CD2, CD5 and CD28, but express CD56 or γδ TCR (Minagawa et al. 2018; Nishimura et al. 2013; Themeli et al. 2015; Vizcardo et al. 2013). Recently, Kaneko’s group modified the T cell differentiation culture and generated iPSC-derived CD8αβ^+^ CD5^+^ CCR7^+^ CD45RA^+^ CD56^−^ T cells with early memory phenotypes, which can give rise to effector T cells for immunotherapies (Kawai et al. 2021). Though the optimized method improved the expression of CD28 and CD62L, the levels of these naïve-associated markers were still lower than primary naïve T cells. During T lymphocyte development in thymus, the interactions of T precursors with signals from thymic epithelial and mesenchymal cells are indispensable (De Smedt et al. 2004; La Motte-Mohs et al. 2005; Rothenberg et al. 2008; Rothenberg and Scripture-Adams 2008). So mimicking T lymphocyte developmental microenvironment in culture systems may generate truly naïve-phenotype T cells in vitro. In 2013, two groups described that human ESC-derived thymic epithelial progenitors (TEPs) can reconstitute thymic microenvironment in thymus-deficient mice (Parent et al. 2013; Sun et al. 2013). The human ESC-derived TEPs were transplanted under the kidney capsule of nude mice to mature into thymic epithelial cells. After 10 weeks of transplantation, CD4^+^ and CD8^+^ single-positive mouse T cells were observed in recipient mice (Parent et al. 2013). Moreover, these TEPs can also support human thymopoiesis in NOD/SCID mice after human HSC transplantation (Gras-Pena et al. 2022; Sun et al. 2013). Then, researchers generated 3D artificial thymic organoids (ATOs) to support positive selection of double positive precursors to mature CD4^+^ or CD8^+^ single-positive T cells using mouse MS5 stromal cell line transduced with human DLL1 in vitro (Montel-Hagen et al. 2019; Seet et al. 2017). These hPSC-derived single-positive T cells possessed TCR-αβ diversity and showed CD45RA^+^ CD45RO^−^ CD27^+^ CCR7^+^ CD1a^low^ phenotype, which was similar to naive T cells from human thymus and blood. Recently, Zeleniak et al. successfully generated human iPSC-derived ATOs for the differentiation of diverse subpopulations of mature T cells in hematopoietic humanized mice (Zeleniak et al. 2022). These above findings suggest that ATOs are essential for T lymphocyte growth from hPSCs in culture, which truly simulate in vivo T cell thymic developmental microenvironment.

In general, the TCRs of hPSC-derived T cells are often rearranged randomly and with no antigen-specificities. It was reported that terminally differentiated T cell-derived iPSCs presented different TCR-β rearrangement patterns identical to those of the original circulating mature T cell clones (Loh et al. 2010; Seki et al. 2010). In 2013, Kawamoto’s group established melanoma antigen MART-1-specific cytotoxic T lymphocytes (CTLs)-derived iPSCs from a melanoma patient. These iPSCs produced CD4^+^ and CD8^+^ double-positive cells and then mature to CD8^+^ single-positive T cells with TCR specific for MART-1 epitope (Vizcardo et al. 2013). After then, researchers successfully generated human immunodeficiency virus-specific CD8^+^ CTLs for acquired immune deficiency syndrome, Epstein-Barr virus (EBV)-specific CTLs for lymphoma, and so on (Ando et al. 2020; Haque et al. 2021; Nishimura et al. 2013). The development of antigen-specific CTLs derived from iPSCs opens up new approaches for therapies for cancer, autoimmune and infective disorders. Recently, Kaneko’s group demonstrated that TCR-engineered iPSCs presented T cell commitment just like antigen-specific CTL-derived iPSCs, which gave an alternative way for antigen-specific CTL production (Iriguchi et al. 2021). Besides CTLs, γδ-T lymphocytes and other types of αβ-T lymphocytes derived from relevant iPSCs were also regenerated recently, such as γ9δ2-T cells, NKT cells, and mucosal-associated invariant T cells (Kitayama et al. 2016; Yamada et al. 2016). However, Kaneko’s group illustrated that the TCR-α of these iPSC-derived T cells was susceptible to be rearranged during double-positive stage in vitro, which resulted in losing of antigen specificity. Then they successfully generated more stabilized and effective TCR-invariable T cells by knocking out a key TCR recombinase gene, RAG2 (Minagawa et al. 2018). In conclusion, these antigen-specific artificial T cells pave the way toward novel immunotherapy for kinds of diseases. However, given the limited available sources of antigen-specific human T cells for iPSC induction, an alternative source of off-the-shelf antigen-targeted hPSCs should be established.

CAR-T immunotherapy is one of the curative therapies for treating hematologic malignancy diseases, solid cancer, and even some autoimmune diseases (Labanieh et al. 2018; Orvain et al. 2021). CAR-T cells are genetically artificial T cells, which can target specific antigens and perform immune killing. Generally, for producing CAR-T cells, enough healthy T cells are collected from mobilized peripheral blood (PB), and then these T cells should be transduced with lentivirus or retrovirus to express CARs (Labanieh et al. 2018). In 2013, Sadelain’s group succeed in generating human CD19-targeted T cells by expressing CARs in healthy T cell-derived iPSCs. Though these iPSC-derived CD19 CAR-T cells displayed innate γδ-T cells phenotypes, they exerted anti-tumor activity in a xenograft model (Themeli et al. 2013). In the following years, several groups successfully regenerated iPSC-originated CAR-T cells with modified differentiation protocols, such as adding SDF1α and p38 inhibitor, educating CAR-T cells with ATOs, and delaying CAR expression during differentiation. They found that using these modified methods, the produced CAR-T cells showed conventional CD8αβ^+^ characteristics and effective anti-tumor function in vivo (Iriguchi et al. 2021). Moreover, Harada et al. engineered LMP1-CAR into LMP2-specific CTL-derived iPSCs. These dual-antigen CAR-T cells inhibited tumor cell growth and persisted long in a mouse lymphoma model, with the potential of mitigating tumor antigen escape (Harada et al. 2022). Up to date, there have been some approaches making CAR-T cells more persistent and enhanced anti-tumor activity in mouse tumor models, like inhibiting TCR-CD3 complex, CD155, β2-microglobulin, and EZH1, or overexpressing IL-15, IL-15R, and antigen E (Eyquem et al. 2017; Jing et al. 2022; Ueda et al. 2023; Wang et al. 2021). All in all, iPSCs can theoretically give rise to enough functional antigen-specific T cells for clinical treatment.

### NK cells

NK cells are essential innate lymphoid cells. Unlike T cells, NK cells exert functions of killing tumor or infected cells in human leukocyte antigen (HLA)-independent manner, thus avoiding graft-versus-host disease (GvHD) after engraftation, which makes NK cells a promising candidate for immunotherapies (Miller et al. 2005; Rubnitz et al. 2010; Ruggeri et al. 2002). The origins of NK cells used in clinical trials include PB, umbilical cord blood (UCB) and immortalized NK cell lines, like NK-92 cell line (Saetersmoen et al. 2019). However, the clinical applications of NK cells are limited by lacked primary NK cells, attenuated functions after cryopreservation, short lifespan in vivo, and so on (Kang et al. 2021; Passweg et al. 2004; Zhu and Kaufman 2019). hPSC-derived NK cells present an alternative avenue for off-the-shelf clinically used NK cells.

In 2005, Kaufman’s group produced ESC-derived NK cells in vitro through sequentially coculturing with two types of stromal cells, with the ability to fight tumor cells (Woll et al. 2005). They identified these hPSC-derived NK cells expressed inhibitory and activating receptors, like KIRs, CD94, NKG2A, NKG2D, NKp30, NKp44, NKp46, and CD16. Further phenotypic analysis showed that the NK cells were a uniform CD94^+^CD117^low/−^ NK cell population which was characterized by a better cytotoxic function and more effective killing abilities than UCB-derived NK cells (Woll et al. 2005, 2009). Since iPSC-derived NK cells were more dependent on Notch signaling of feeder cells, using DLL1 and DLL4 genetically engineered stromal cells could also help NK cell commitment and expansion (Mesquitta et al. 2019; Zeng et al. 2017). Another classical NK cell differentiation method was related to EB formation and clone 9.mbIL-21 artificial antigen-presenting cell stimulation. The membrane-bound IL-21 could expand enough functional NK cells for therapy from fewer than 250,000 hPSCs (Hermanson et al. 2016; Ni et al. 2011; Woll et al. 2009). Actually, Cichocki et al. demonstrated that by coculture of iPSC-NK cells and irradiated K562 cells transduced with IL-21 and 4-1-BB ligand, the generated NK cells were transcriptionally similar to PB-derived NK cells and cooperated with T cells and anti-PD-1 therapy in vivo (Cichocki et al. 2020).

In addition, hPSCs can also be engineered through knock in or out some key genes to modify hPSC-derived NK cells for allogeneic therapies. On the one hand, to enhancing persistence and cytotoxicity of iPSC-NK cells, several groups established non-cleavable CD16A-iPSCs-generated NK cells by mutating CD16A gene in iPSCs (Blum et al. 2016; Cichocki et al. 2022; Jing et al. 2015; Snyder et al. 2018; Zhu et al. 2020b). These engineered iPSC-derived NK cells combined with specific monoclonal antibodies showed high affinity to tumor cells than primary NK cells and unmodified NK cells in different mouse models (Blum et al. 2016; Zhu et al. 2020b). Also, there is a CD16-engineered iPSC-derived NK cell product, FT516 (ClinicalTrials.gov: NCT04023071), has been produced. Besides, knocking out CISH also improved iPSC-NK cells’ persistence and cytotoxicity in vivo via enhancing IL-15 pathway (Zhu et al. 2020a). Recently, Woan et al. had been successfully produced a triple-gene-edited (expressing non-cleavable CD16 and IL15-RF, knocking out CD38) iPSC-NK cells that elicited strong anti-tumor activity in vivo (Woan et al. 2021). On the other hand, to increase the iPSC-NK cells’ specificity, Kaufman’s group generated CAR-engineered iPSC-derived NK cells and showed improved survival compared with antigen-specific CAR-T cells in a murine ovarian cancer model (Li et al. 2018). Interestingly, there have been several iPSC-derived NK cell products engineered with multiple functional modalities, like FT596 (CD19 CAR, expressing non-cleavable CD16 and IL15-RF) (ClinicalTrials.gov: NCT04245722), FT576 (BCMA CAR, expressing non-cleavable CD16 and IL15-RF, knocking out CD38) (ClinicalTrials.gov: NCT05182073).

Finally, compared with iPSC-derived CAR-T cells, there are several advantages of iPSC-derived CAR-NK cells: (1) The application of iPSC-NK cells does not need HLA-matching between donors and recipients, which may make a truly off-the-shelf product. (2) Given the lacking of HLA-dependent activation, NK cell therapy has less associated toxicities and GvHD, especially in an allogeneic condition. (3) CAR-NK cells kill tumor cells also in a CAR-independent manner, such as CD16-mediated ADCC (Xie et al. 2020). All in all, hPSC-derived NK cells have a great clinical application prospect.

### B cells

During B lymphopoiesis in BM, HSCs firstly differentiate into common lymphoid progenitors and then develop into early-B cells with features of DJ rearrangement in height (H) chain as well as expression of Igα and VpreB in cytoplasm. After then, accompanied by VDJ rearrangement in H chain and activation of B lineage marker CD19, early-B cells develop into pro-B cells. Sequentially, cytoplasmic µ-H chain rearrangement makes pro-B into the pre-B stage, with the various surface µ-H chains associated with temporary L chains called pre-B cell receptor (BCR). Immature B cells specifically express IgM in the surface, which is also called BCR, consisting of µ-H chains associated with κ or λ-L chains. Finally, these immature B cells migrate to spleen for the development of various B cell subsets (LeBien 2000).

It was reported that human ESCs present B lineage potential in OP9 coculture differentiation process, which marked the definitive hematopoiesis (Vodyanik et al. 2005). In 2011, Carpenter et al. produced iPSC-derived B cells by coculturing with OP9 cell line and MS5 cell line sequentially and showed these iPSC-originated CD45^+^ CD19^+^ CD10^+^ B cells did not express surface IgM and CD5, which presented pre-B cell identity (rearrangement of DJ regions in H chain) (Carpenter et al. 2011). Then this group found that iPSC-derived CD144^+^ CD73^−^ CD235a^−^/CD43^−^ HE was able to generate B cells expressing IgM in cell surface, which are immature-B phenotypes (rearrangement of VDJ regions in H chain). Further transcriptome profiling showed high similarity between iPSC-derived B cells and UCB-derived counterparts (French et al. 2015). However, given B cells consist of B-1 cells, marginal zone (MZ) B cells, and follicular (FO) B cells, one group demonstrated that mouse ESC-derived B cells possessed incomplete populations, with B-1 and MZ subsets but not FO subset (Lin et al. 2019). Most recently, Zhang et al. successfully generated mouse ESC-derived B cells with B-1, MZ, and FO subpopulations, via expressing Lhx2, Hoxa9, and Runx1 in mouse ESCs. The regenerative B cells showed a similar trajectory of B lymphopoiesis and could reconstitute adaptive humoral immune responses in B lineage-deficient mice (Zhang et al. 2022). Although B cells are refractory to be reprogrammed into iPSCs, researchers have yet successfully generated PB or UCB-derived mature B cell-reprogrammed iPSCs carrying complete VDJ rearrangement in H chain (Bueno et al. 2016; Munoz-Lopez et al. 2016). Munoz-Lopez et al. found that FL-derived B progenitors were easier to be reprogrammed into iPSCs, though with less immunoglobulin gene rearrangement compared with mature B cell derivation (Munoz-Lopez et al. 2016). Interestingly, EBV-immortalized B cell lines, isolated from patients, can also be reprogrammed to iPSCs, though these iPSCs may lose EBV-related elements in generation (Choi et al. 2011a, b; Rajesh et al. 2011).

In conculsion, the regeneration of functional B cells from hPSCs in vitro still remains challenging, and the existing scarce protocols only produce incomplete B cell subgroups with defective functions. Additionally, B cells are refractory to reprogramming, which makes B cell regeneration more difficult.

## Directed differentiation of megakaryocytic and erythroid cells from hPSCs

### MKs and platelets

Platelets are the smallest non-nucleated blood cells released from polyploidy MKs in the BM. In 2006, Gaur and colleagues achieved the effective differentiation of MKs from human ESCs for the first time, and these cells were capable of producing functional platelets (Gaur et al. 2006). In general, MKs and platelets could be generated from hPSCs through a three-step culture process, including the generation of HSPCs, MKs, and platelets. By using the approaches shown in Fig. 2, hPSCs differentiate into HSPCs in the first stage. Based on this, MKs and platelets were obtained under conventional culture methods with SCF, TPO, FLT-3L, and IL-11 (Gaur et al. 2006). An ideal culture condition for hPSC-derived MKs should be able to generate large numbers of functional platelets. Coculturing with feeder cells is a common strategy for enhancing hematopoiesis (Table 1). OP9 and C3H10T1/2 stromal cells were used to support the megakaryopoiesis and thrombopoiesis (Gaur et al. 2006), but the efficiency was still low with these feeder cells, suggesting it is not enough to improve megakaryopoiesis just by mimic microenvironment. Manipulating essential genes involved in megakaryopoiesis and thrombopoiesis could benefit the improvement of the production of platelets (Takayama et al. 2008; Takayama et al. 2010). For instance, Eto’s group established immortalized MK progenitor cell line from hPSC-derived hematopoietic progenitors by introducing doxycycline-inducible MYC, BMI1, and BCL-XL, capable of producing over 10^11^ platelets within 5 days (Nakamura et al. 2014). Recently, they increased the induction efficiency of this progenitor cell line proliferation for 10^12^ fold by knockdown of CDKN1A and p53 (Sone et al. 2021). Besides, TFs like GATA1, FLI1, and TAL1 were also manipulated to improve the production efficiency of MKs (Evans et al. 2021; Moreau et al. 2016). Based on these, the feeder-free condition was well established to generate clinical-grade platelets in numerous studies (Liu et al. 2015; Moreau et al. 2016). However, in these 2D culture systems, although the yield of functional platelets was high, the generation of platelets per MKs was very low (5–15 per MKs) and was far short of the production in vivo (200–7700 platelets per MKs in human body and 500–1000 platelets per MKs in mouse body (Lefrancais et al. 2017). Recently, bioreactors showed great potential in increasing the production of hPSC-derived platelets (Evans et al. 2021; Moreau et al. 2016). Eto’s group developed a turbulence-controlled bioreactor that can produce 100 billion-order platelets from human iPSC-MKs, increasing the production of platelets from 14 to 70–80 per MKs (Ito et al. 2018), suggesting combining gene manipulation with bioreactor is a more efficient strategy for iPSC-derived MKs and platelets.

In addition, to prevent platelet transfusion failure caused by mismatched HLA, universal platelets are produced. Under serum/Xeno/feeder-free conditions, Feng et al. obtained universal platelets (HLA-ABC negative) from human iPSCs. These platelets resembled blood platelets in morphology and function (Feng et al. 2014). Several teams generated universal platelets from hPSCs knocked out HLA class I molecules by CRISPR/Cas9 technology (Feng et al. 2014; Norbnop et al. 2020; Suzuki et al. 2020). Thus, how to produce large-scale hPSC-derived universal platelets under clinical-grade level in vitro could be a new challenge.

### RBCs

RBCs are one of the most important cell types for delivering oxygen. Scientists from Addenbrooke’s Hospital in Cambridge have transfused lab-made RBCs into a human volunteer in a world-first trial in 2022, laying the groundwork for manufacturing RBCs that can safely be used for clinical application. By coculturing the mouse BM stromal cell line S17 and the YS endothelial cell line C166, erythroid progenitor cells were successfully generated from human ESCs in 2001 (Kaufman et al. 2001). In 2002, Douay’s team developed a feasible approach for harvesting a sizable amount of erythroid cells from UCB CD34^+^ cells (Neildez-Nguyen et al. 2002). Since then, numerous erythroid cells generated from hPSCs have also been successfully produced (Lapillonne et al. 2010; Sun et al. 2018).

In general, there are two steps for differentiation of hPSCs into erythrocytes, including the generation of HSPCs (CD34^+^ cells) and mature erythrocytes (Ebrahimi et al. 2020; Sun et al. 2018). CD34^+^ cells differentiate into erythroid cells under effective culture methods with SCF, IL-3, erythropoietin (EPO), dexamethasone, insulin, heparin, and transferrin (Chen et al. 2022; Kobari et al. 2012; Wang et al. 2022). Stromal cells, such as OP9 (Dias et al. 2011; Klimchenko et al. 2009; Trakarnsanga et al. 2018) and stromal cells generated from FL (Ma et al. 2008), were usually applied to imitate the microenvironment in vitro and enhance the generation of erythroid cells. Type of hemoglobins reflects the oxygen-carrying capacity of RBCs, however, most hPSC-derived erythroblasts from different differentiation methods express fetal hemoglobins (ζ, ɛ, γ), suggesting a primitive hematopoietic origin. By coculturing human ESCs with mouse FL-derived stromal cells, Ma et al. produced massive erythroid cells. These erythroid cells with adult β-globin expression had a normal oxygen-carrying capacity, glucose-6-phosphate dehydrogenase activity, and effectively enucleate (Ma et al. 2008). Furthermore, they obtained erythroblasts expressing β-globin (around 90%) by coculturing human ESCs with AGM-S3 (Mao et al. 2016). Since FL and AGM are important sites for definitive hematopoiesis, suggesting the microenvironment of the definitive site benefits the expression of β-globin. Stromal-free culture systems are another strategy to produce erythrocytes from hPSCs, but almost all of these erythrocytes express fetal globins (Lapillonne et al. 2010; Lu et al. 2008), which may be because of a lack of definitive microenvironment signalings. Additionally, stromal cells have been shown in several investigations to considerably enhance the enucleation of hPSC-derived erythroblasts (Lu et al. 2008; Shen et al. 2020). Douay’s group showed that the number of enucleated erythrocytes (expressing fetal globins) generated from iPSCs obtained from human fetal and adult fibroblasts is less than those from human ESC (Lapillonne et al. 2010). Besides, a report showed that although erythroid cells generated from different sources of iPSCs (UCB-, PB-, and BM-derived) showed similar hemoglobin (fetal type) and oxygen-carry ability, discrepancies in differentiation and maturation efficiencies which UCB-derived iPSCs generated the moistest enucleated erythrocytes (Cho et al. 2023). Those studies suggest that the origin of iPSCs is also important for generating enucleating erythrocytes. Thus, the mechanism of how microenvironment influences erythroid differentiation and maturation may be a breakthrough for producing β-globin-expressed and enucleated erythrocytes in vitro. Bioreactors are used for erythrocyte production by mimicking the in vivo environment physically. Studies using bioreactors showed large quantities of UCB-derived erythroid cells while being rarely used on hPSC-derived (Elvarsdottir et al. 2020; Lee et al. 2015). In a 50 mL 3D culture system, 0.85 billion erythroblasts were generated from O-negative human iPSCs and around 60% of enucleated erythrocytes were obtained when cocultured with OP9, although these cells still expressed fetal globins (Sivalingam et al. 2021). In addition, small molecules, such as SR1 (Antagonist of aryl hydrocarbon receptor) and IBMX (Inhibitor of cAMP and cGMP phosphodiesterase), were frequently utilized in feeder-free cultures to supply the erythroid differentiation (Chen et al. 2022; Olivier et al. 2016). Therefore, combining a 3D culture system with small molecules may achieve large-scale production.

hPSC-derived erythroblasts have similar properties to UCB-derived erythroblasts, such as oxygen-carrying and deformability, pointing to potential clinical use in blood transfusion (Kobari et al. 2012; Lu et al. 2008; Mazurier et al. 2011). However, low β-globin expression and enucleation ratio in hPSC-derived erythroblasts are indications that these erythroblasts are not fully mature RBCs (Bernecker et al. 2019; Chen et al. 2022; Dorn et al. 2015; Wang et al. 2022; Xu et al. 2022). Wang et al. compared orthochromatic derived from human ESCs and UCB and provided insights into the limited expansion and impaired enucleation of ESC-derived erythroblasts (Wang et al. 2022), but the mechanisms remained lacking. Exploring the mechanisms of impaired enucleation of ESC-derived erythropoiesis is needed for improving efficiency of ESC-derived RBCs in vitro.

## Directed differentiation of myeloid cells from hPSCs

### Monocytes and macrophages

Macrophages play an essential role in tissue homeostasis and innate immunity. Different hematopoietic sites, such as the YS, FL, and BM, give rise to different types of macrophages (Dzierzak and Bigas 2018; Guerriero 2018). For instance, like microglia, tissue-resident macrophages develop from HSC-independent progenitors which originated from the YS and FL (Ginhoux et al. 2010). Unlike tissue-resident macrophages, monocyte-derived macrophages develop from HSCs in BM, which populates tissues only under inflammatory conditions (Serbina et al. 2008).

Several groups have developed systems to differentiate hPSC into macrophages. Coculture or EB systems were used to obtain hematopoietic progenitors, and IL-3 and macrophage colony-stimulating factor (M-CSF) were used to differentiate these progenitors into macrophages (Lachmann et al. 2015; van Wilgenburg et al. 2013). Large numbers of monocytes were yielded from hPSCs under feeder- and serum-free conditions in IL-3 and M-CSF (Karlsson et al. 2008). These monocytes could differentiate into mature macrophages and display the typical characteristics, such as a high level of phagocytosis, the release of specific cytokines in response to lipopolysaccharide, and production of a pro-inflammatory cytokines profile upon activation (van Wilgenburg et al. 2013). Kaufman’s group developed a feeder/serum-free method to rapidly produce large numbers of macrophages from iPSCs (approximately 4 × 10^6^ cells for 4 weeks from 1 well of a 6-well plate). These macrophages exhibited similar surface cell markers and phagocytic activity to BM-derived macrophages. Additionally, in an immunodeficient mouse model, these iPSC-derived macrophages reduced inflammation and improved liver fibrosis (Bernareggi et al. 2019; Pouyanfard et al. 2021). Besides, hPSCs also offer a cell source for the production of tissue-resident macrophages (Lee et al. 2018), and these cells showed a tissue-specific functional capacity like immune responses and tissue homeostasis (Ackermann et al. 2018, 2022; Xu et al. 2020). In addition, iPSC-derived macrophages have been utilized in several studies to treat cancer. Ikeda et al. established an iPSC-derived myeloid/macrophage cell line to rapidly generate massive macrophage-like cells. Intraperitoneal administration of these iPSC-derived myeloid/macrophages into mice with pre-established peritoneal NUGC-4 tumors resulted in massively accumulating and infiltrating into the tumor tissue and inhibiting tumor growth (Koba et al. 2013). Besides, iPSC-derived macrophage cells with CAR-expressing also showed great potential in immunotherapy. Both CD19 CAR-macrophage and mesothelin CAR-macrophage showed the ability to phagocytose CD19-expressing K562 leukemia cells and increased phagocytosis activity against mesothelin-expressing OVCAR3/ASPC1 ovarian/pancreatic cancer cells, respectively. Additionally, mesothelin CAR-macrophage demonstrated reduced tumor burden in vivo (Zhang et al. 2020). Given the broad field of applied macrophage research, advanced differentiation approaches are needed to produce application-specific macrophages.

### Granulocytes

Generation of granulocytes from hPSCs requires hematopoietic progenitor development followed by myeloid differentiation. Compared to other myeloid cell types, differentiation from hPSCs to granulocytes has received less attention. Lachmann et al. reported an EB-based differentiation method can yield about 0.5 × 10^6^ granulocytes weekly (Lachmann et al. 2015). Different combinations of cytokines, such as granulocyte colony-stimulating factor (G-CSF) for neutrophils, IL-5 for eosinophils, and IL-3 for basophils, are better suited for a specific type of granulocytes (Hansen et al. 2019; Sweeney et al. 2016).

Neutrophils are essential to respond to bacterial and fungal infections. Several groups have reported methods to generate hPSC-derived neutrophils, and these neutrophils fulfilled important functions, such as engaging in bactericidal activity and chemotaxis, they also displayed a unique gene expression profile compared to PB neutrophils (Brok-Volchanskaya et al. 2019; Choi et al. 2009, 2011a, b; Yokoyama et al. 2009). However, these methods wherein obtaining neutrophils from hPSCs took more than 14 days. Recently, functional neutrophil-like cells at a clinically applicable scale were generated from human iPSC-derived neutrophil-primed progenitors by over-expressing c-MYC, BMI1, and BCL-XL. Moreover, neutrophils were quickly generated within 4 days and these cells could rapidly against lethal bacterial infections in vivo (Miyauchi et al. 2021). Rare reports of eosinophils or basophils produced from hPSCs exist. Deng’s lab reported a chemically defined method to induce hPSC differentiating into eosinophils (around 1000 eosinophils were generated from a single human ESC), and these eosinophils showed similar features with naïve eosinophils at the transcriptional level, and competent tumor-killing capacity both in vitro and in vivo (Lai et al. 2021). However, this method needs 32 days to obtain eosinophils from human ESCs.

Mast cells play an important role in common immunological disorders, such as allergies and asthma. There are 2 types of mast cells: connective tissue and mucosal type. Mast cells generated from hPSCs display characters of connective tissue-type mast cells, such as responding to various allergens, however, the differentiation processes take a very long time (8–12 weeks) (Bian et al. 2021; Ikuno et al. 2019; Kovarova et al. 2010; Yamaguchi et al. 2013). In 2018, Dzierzak’s group developed a rapidly efficient method to generate mast cells from hPSCs in 12 to 16 days by sorting GATA2^+^ cells (Kauts et al. 2018), but it is not suitable for large-scale production. Recently, Luo et al. established a 4-step method for generating phenotypically mature, functional mast cells from hPSCs (5 × 10^5^ mast cells from 40 hPSC colonies) under a feeder-free condition, and the time of differentiation was reduced to 52 days (Luo et al. 2022). hPSC-derived mast cells can be used as a novel testing system for allergens in clinical (Igarashi et al. 2018). Thus methods of generating granulocytes with less time-consuming and higher efficiency are needed.

## Major challenges and potential solutions

hPSCs have an ability of unlimited proliferation, which makes their enormous potential for cell therapy. However, hPSC-derived products exist practical issues with tumorigenicity, immunogenicity, and heterogeneity that limit the clinical application, which is discussed in detail by Yamanaka (Yamanaka 2020). Because residual stem cells may result in tumors in vivo, high-purity blood cells are needed for in vitro culturing. Numerous groups have produced mature blood cells with high purity (> 95%), such as NK cells (Cichocki et al. 2020), T cells (Montel-Hagen et al. 2019), and platelets (Nakamura et al. 2014), indicating low tumorigenicity *in vivo.* Additionally, hPSCs harboring suicide genes may increase the safety of hPSC-derived products (de Luzy et al. 2021). HLA-matching is critical for efficient cell therapy. Although autologous iPSCs avoid HLA-matching problems, they need higher expenses on monetary value and time. Therefore, hPSC-derived universal blood cells offer a new strategy. hPSCs do not express HLA-ABC which was used to generate universal blood cells such as platelets (Feng et al. 2014), NK cells (Saetersmoen et al. 2019), and T cells (Guo et al. 2020). Some hPSC-derived blood cells have reached or are about to reach clinical trials, like NK cells (ClinicalTrials.gov: NCT03841110; ClinicalTrials.gov: NCT04106167); platelets (Trial ID: jRCTa050190117). Despite the low efficiency of hPSC-derived RBCs, mature RBCs are not tumorigenic and immunogenic because they do not have a nucleus and express HLA antigens, suggesting hPSC-derived RBCs have a huge potential for clinical use (Bernecker et al. 2019).

On the other hand, it is necessary to reduce additional risks and process-related difficulties related to the generation of mature blood cells in vitro. Exon- or tumor-derived feeders and serum were used to improve the efficiency and maturity of blood cells in many methods, like OP9 (Trakarnsanga et al. 2018) and K562 (Cichocki et al. 2020). However, producing mature blood cells from hPSC in a clinic-appropriate system is still a low-efficient process, particularly for generating HSCs and RBCs. Therefore, the challenge for large-scale production of hPSC-derived mature blood cells in exon-free systems needs to be overcome. Bioreactors are used to mimic the physical environment in vivo, which may play an essential role in the maturation of blood cells. Combining several technologies (such as biosynthesis, gene editing and biomaterial) with bioreactors could help break through the hurdles of hPSC-derived blood cell maturity.

## Conclusions

hPSCs not only provide a tool for studying the development of human embryo hematopoiesis, but also an infinite source of blood cells (Bernareggi et al. 2019; Ivanovs et al. 2017). In past decades, several groups have developed many efficient methods that obtain a considerable output of blood cells from hPSCs, while it has been difficult to generate mature cells in vitro. In the next stage, researches focusing on the maturation of hPSC-derived blood cells will increase markedly in the coming years. Additionally, other challenges like serum- and exon-free conditions and cost-effective production of mature cells need to be overcome before patients benefit from hPSC-based cell therapy (Ebrahimi et al. 2020; Harding and Mirochnitchenko 2014; Pratumkaew et al. 2021). Furthermore, efficient, simple, and defined methodologies are required to develop good manufacturing practice standards for hPSC-derived blood cells, facilitating the translation of these products into the clinic (Doss and Sachinidis 2019).

## Data Availability

Not applicable.

## Abbreviations

YS: Yolk sac
MKs: Megakaryocytes
EMPs: Erythro-myeloid progenitors
HSCs: Hematopoietic stem cells
LT-HSCs: Long-term HSCs
HE: Hemogenic endothelium
AGM: Aorta-gonad mesonephros
EHT: Endothelial-to-hematopoietic transition
FL: Fetal liver
BM: Bone marrow
RBC: Red blood cells
CAR: Chimeric antigen receptor
hPSCs: Human pluripotent stem cells
ESCs: Embryonic stem cells
iPSCs: Induced pluripotent stem cells
NK: Natural killer
HSPCs: Hematopoietic stem/progenitor cells
EBs: Embryoid bodies
3D: Three-dimensional
BMP: Bone morphogenetic protein
bFGF: Basic fibroblast growth factor
VEGF: Vascular endothlial growth factor
SCF: Stem cell factor
TPO: Thrombopoietin
FLT-3L: FMS-related tyrosine kinase 3 ligand
IL-3: Interleukin-3
SR1: StemRegenin 1
2D: Two-dimensional
TFs: Transcription factors
TCR: T cell receptor
TEPs: Thymic epithelial progenitors
ATOs: Artificial thymic organoids
CTLs: Cytotoxic T lymphocytes
EBV: Epstein-Barr virus
PB: Peripheral blood
HLA: Human leukocyte antigen
GvHD: Graft-versus-host disease
UCB: Umbilical cord blood
BCR: B cell receptor
MZ: Marginal zone
FO: Follicular
EPO: Erythropoietin
M-CSF: Macrophage colony-stimulating factor
G-CSF: Granulocyte colony-stimulating factor
