# Correction: Generating hematopoietic cells from human pluripotent stem cells: approaches, progress and challenges

**DOI:** 10.1186/s13619-023-00177-4

**Published:** 2023-10-09

**Authors:** Haiqiong Zheng, Yijin Chen, Qian Luo, Jie Zhang, Mengmeng Huang, Yulin Xu, Dawei Huo, Wei Shan, Ruxiu Tie, Meng Zhang, Pengxu Qian, He Huang

**Affiliations:** 1 Bone Marrow Transplantation Center, the First Affiliated Hospital, Zhejiang University School of Medicine, Zhejiang University, Hangzhou, 310012 China; 2 Liangzhu Laboratory, Zhejiang University Medical Center, 1369 West Wenyi Road, Hangzhou, 311121 China; 3 Institute of Hematology, Zhejiang University, Hangzhou, 310012 China; 4 Zhejiang Province Engineering Laboratory for Stem Cell and Immunity Therapy, Hangzhou, 310012 China; 5 Center for Stem Cell and Regenerative Medicine and Bone Marrow Transplantation Center of the First Affiliated Hospital, Zhejiang University School of Medicine, Hangzhou, 310058 China

**Correction: Cell Regen 12, 31 (2023)**

**https://doi.org/10.1186/s13619-023-00175-6**

Following publication of the original article (Zheng et al. 2023), the authors reported that Figs. 1 and 2 were in the wrong order. The correct Figs. 1 and 2 have been provided in this Correction.

**Fig. 1:**
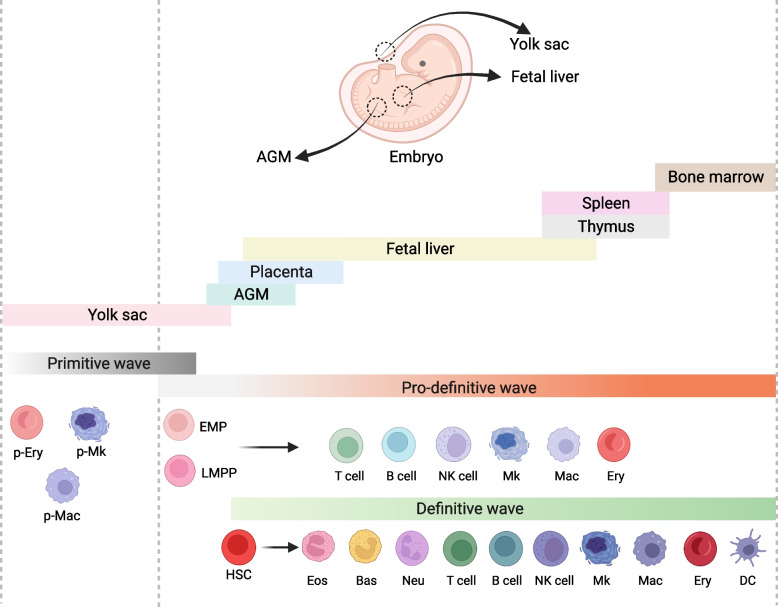
Schematic representations of embryonic hematopoiesis in vivo. There are 3 waves of hematopoietic cell generation, including primitive, pro-definitive, and definitive HSC. Among them, the primitive hematopoietic cells, including erythrocytes, megakaryocytes and macrophages, emerge in the extraembryonic yolk sac blood islands; the pro-definitive wave of hematopoiesis occurs in the yolk sac and generates definitive erythro-myeloid progenitors (EMPs) and lymphoid-primed progenitors (LMPPs) in the yolk sac; the definitive HSCs arise in the intraembryonic aorta–gonad–mesonephros region (AGM) and migrate into the fetal liver for maturation, expansion and differentiation, then initiate mobilization to the bone marrow to supply life-long hematopoiesis

**Fig. 2:**
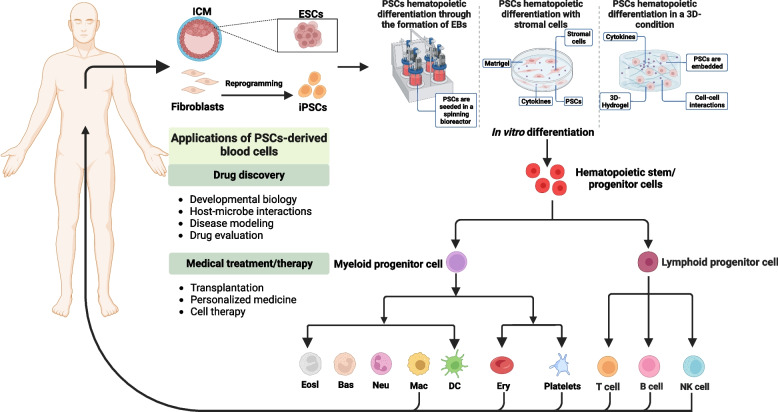
Overview of current methods of inducing PSCs into blood cells in vitro and potential applications of PSCs-derived blood cells. Several differentiation methods have been developed to derive blood cells from PSCs in vitro at present: through the formation of EBs, co-culture with stromal cells and induced hematopoietic differentiation in a 3D-condition. The generated blood cells could be utilized in certain fields: (1) Drug discovery: developmental biology, host-microbe interactions, disease modeling and drug evaluation; (2) Medical treatment/therapy for clinical use, such as transplantation, personalized medicine and cell therapy

The original article (Zheng et al. 2023) has been corrected.

## References

[CR1] Zheng H, Chen Y, Luo Q (2023). Generating hematopoietic cells from human pluripotent stem cells: approaches, progress and challenges. Cell Regen.

